# Preliminary analysis of the expression of ZBTB1 in human pancreatic carcinoma

**DOI:** 10.1111/jcmm.16804

**Published:** 2021-07-27

**Authors:** Ming‐yang Cheng, Yan Zeng, Yu Sun, Chun‐wei Shi, Jun‐hong Wang, Feng‐di Li, Yi‐yuan Lu, Jing‐Ying Wang, Ru‐Yu Wang, Xin‐yang Li, Xiao‐xu Li, Shu‐hui Fan, Gui‐lian Yang, Xin Cao, Bin Xu, Chun‐feng Wang

**Affiliations:** ^1^ College of Veterinary Medicine Jilin Agricultural University Changchun China; ^2^ Jilin Provincial Engineering Research Center of Animal Probiotics Jilin Agricultural University Changchun China; ^3^ Key Laboratory of animal production and product quality safety of Ministry of Education Jilin Agricultural University Changchun China; ^4^ Center for Biomedical Research College of Life Science and Engineering Northwest Minzu University Lanzhou China; ^5^ Department of General Surgery Shanghai 10th People's Hospital Tongji University School of Medicine Shanghai China

## CONFLICT OF INTEREST

The authors confirm that there are no conflicts of interest.

## AUTHOR CONTRIBUTION

**Ming‐yang Cheng:** Formal analysis (lead); Investigation (lead); Methodology (equal); Project administration (equal); Writing‐original draft (equal). **Yan Zeng:** Data curation (equal); Investigation (equal); Methodology (equal); Validation (equal). **Yu Sun:** Investigation (supporting); Methodology (supporting). **Chun‐wei Shi:** Investigation (supporting); Methodology (supporting). **Jun‐hong Wang:** Investigation (supporting); Methodology (supporting). **Feng‐di LI:** Investigation (supporting); Methodology (supporting). **Yi‐yuan Lu:** Investigation (supporting); Methodology (supporting). **Jing‐ying Wang:** Investigation (supporting); Methodology (supporting). **Ru‐yu Wang:** Investigation (supporting); Methodology (supporting). **Xin‐yang Li:** Investigation (supporting); Methodology (supporting). **Xiao‐xu Li:** Investigation (supporting); Methodology (supporting). **Shu‐hui Fan:** Investigation (supporting); Methodology (supporting). **Gui‐lian Yang:** Project administration (equal); Resources (equal); Supervision (equal). **Xin Cao:** Conceptualization (equal); Funding acquisition (equal); Project administration (equal); Supervision (equal); Writing‐original draft (lead); Writing‐review & editing (equal). **Bin Xu:** Conceptualization (equal); Investigation (equal); Methodology (lead); Project administration (equal); Writing‐review & editing (equal). **Chunfeng Wang:** Conceptualization (lead); Funding acquisition (lead); Supervision (lead); Writing‐review & editing (equal).

Dear Editor,

The transcriptional repressor Zinc Finger and BTB domain‐containing protein 1 (ZBTB1) is an essential member of the BTB‐ZF family and plays an important role in the development of the immune system and DNA repair.^1^ The mechanisms by which ZBTB1 regulates multiple immune‐related pathways involved in cancer progression have been investigated.^2^ Although ZBTB1 has been indicated to be a tumour suppressor in breast cancer, its biological functions and clinical significance in other malignant tumours remain unclear.^3^ Pancreatic carcinoma is a fatal malignant tumour with an increasing incidence. Due to difficulty in early diagnosis and poor prognosis, the 5‐year survival rate is only 2%–9%.^4^ Therefore, it is very important to better understand the associated risk genes and prognostic indicators of pancreatic carcinoma. To our knowledge, bioinformatics analysis has not been applied to explore the role of ZBTB1 in pancreatic carcinoma. Our study revealed a candidate driver of pancreatic carcinoma that may be a potential target for precision treatment.

To evaluate the clinical significance of ZBTB1 in pancreatic carcinoma, we used human tissue microarray (TMA) technology and immunohistochemistry (IHC) with an anti‐ZBTB1 monoclonal antibody to examine 60 tumour tissue samples and 47 matched and unmatched non‐neoplastic tissue samples. The IHC results were assigned an average score. Brown or brownish‐yellow particles were defined as positive cells. First, the staining intensity was characterized as follows: no staining, 0 points; pale yellow staining, 1 point; brownish‐brown staining, 2 points; and brown staining, 3 points. Then, the percentage of stained cells relative to the total cells was classified as follows: ≤5%, 0 points; 6%–25%, 1 point; 26%–50%, 2 points; 51%–75%, 3 points; and ≥76%, 4 points. The score of each sample was calculated as the sum of the scores of (1) and (2). A score ≥8 was regarded as high expression, and a score <8 was regarded as low expression.^3^, ^5^

**FIGURE 1:**
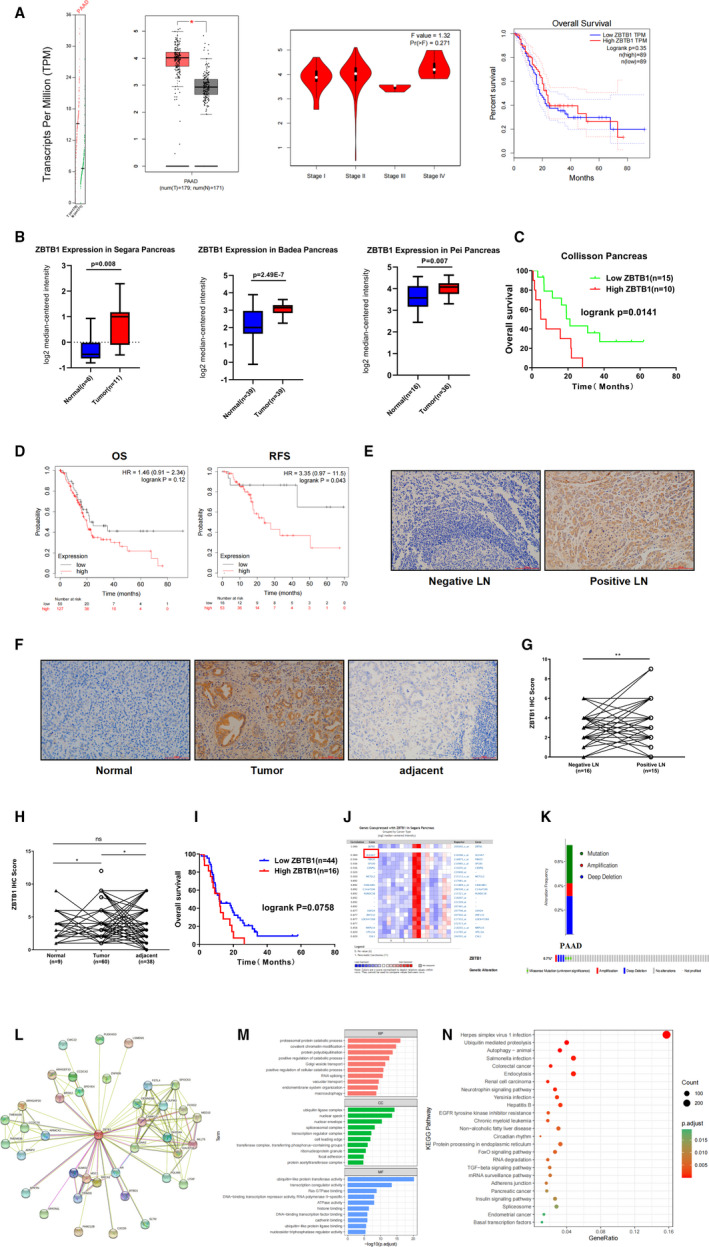
High expression of ZBTB1 in human pancreatic carcinoma promotes malignant tumour progression. (A) Expression of ZBTB1 and its function in pancreatic carcinoma (GEPIA). B) The transcription levels of ZBTB1 in pancreatic carcinoma (Oncomine). (C) Kaplan‐Meier curve showing the overall survival of 25 patients with pancreatic carcinoma in the Collision pancreas dataset (Oncomine). (D) The prognostic value of the mRNA level of ZBTB1 in pancreatic carcinoma patients (Kaplan‐Meier Plotter). (E) Representative IHC images (200×) showing ZBTB1 protein expression in patients negative for lymph node metastasis (left) and positive for lymph node metastasis (right). (F) Representative IHC images (200×) showing ZBTB1 protein expression in normal pancreatic tissues (left), PDAC (middle) and carcinoma‐adjacent tissues (right). (G and H) Statistical analysis of ZBTB1 expression according to the IHC score. I Kaplan‐Meier curve showing the overall survival of 60 patients with pancreatic carcinoma. J ZBTB1 gene expression and mutations in pancreatic carcinoma (cBioPortal). (K) Genes co‐expressed with ZBTB1 in the Sergara Pancreas dataset (Oncomine). (L) ZBTB1 protein interaction network (String‐DB). (M‐N) The functions of ZBTB1 and genes significantly correlated with ZBTB1. The data were analysed and processed by GraphPad Prism 8.0 software. Student's *t* test was used to compare the data from the two groups. The *symbol indicates *p* < 0.05, **indicates *p* < 0.01 and ***indicates *p* < 0.001

In this study, the mRNA expression of ZBTB1 between pancreatic carcinoma and pancreatic tissue was compared by GEPIA (Gene Expression Profiling Interactive Analysis) (http://gepia.cancer‐pku.cn/). The results indicated that the expression levels of ZBTB1 were higher in pancreatic carcinoma tissues than in normal tissues, and the expression of ZBTB1 did not significantly differ based on the tumour stage of pancreatic carcinoma. The Kaplan‐Meier curve and log rank test analyses revealed that the increased ZBTB1 mRNA levels were not significantly associated with the overall survival (OS) of all of the patients with pancreatic carcinoma (*p* = 0.35) (Figure 1A).

To validate the above conclusions, we next used the Oncomine Gene Expression Array database (https://www.oncomine.org/) to analyse the ZBTB1 mRNA expression level and prognosis of patients with pancreatic carcinoma.^6^ In 3 data sets with a total sample size of 147, ZBTB1 was overexpressed to some extent in pancreatic carcinoma tissues compared with normal tissues (Figure 1B). ZBTB1 was found to be more highly expressed in pancreatic carcinoma (*p* = 0.008), pancreatic ductal adenocarcinoma (*p* = 2.49E‐7) and pancreatic carcinoma (*p* = 0.007) samples than in normal samples. Remarkably, using clinical samples in Collision's datasets to analyse OS, we did not come to a similar conclusion. The mRNA level of ZBTB1 was significantly correlated with OS, and the mortality of patients with high expression was higher (*p* = 0.0141)(Figure 1C). The effect of ZBTB1 on the survival of pancreatic carcinoma patients was further explored by the Kaplan‐Meier Plotter tools (https://kmplot.com/analysis/). Kaplan‐Meier curve and log rank test analyses revealed that increased ZBTB1 mRNA levels were significantly associated with relapse‐free survival (RFS) (*p* = 0.043) but not overall survival (OS) (*p* = 0.12) in all of the patients with pancreatic carcinoma (Figure 1D).

Figure 1E‐F shows that ZBTB1 was aberrantly expressed in pancreatic carcinoma and positive in lymph node metastasis (Figure 1E‐F). The IHC score results showed that ZBTB1 protein expression in the positive lymph node metastasis group was significantly higher than that in the negative lymph node metastasis group (Figure 1G); the expression level in pancreatic carcinoma tissue was significantly higher than that in adjacent normal tissues (Figure 1H). This indicates that ZBTB1 is necessary to maintain the malignant phenotype and for tumour metastasis. Meanwhile, the survival curve results showed that high ZBTB1 expression was not significantly associated with overall survival (OS) (*p* = 0.0758) (Figure 1I). Overall, in a previous study, we found that ZBTB1 was highly expressed in pancreatic carcinoma and significantly associated with patient RFS but not OS.

Obviously, the expression of ZBTB1 is clearly related to the occurrence of pancreatic carcinoma. We decided to use the cBioPortal online tool (cBioPortal for Cancer Genomics) (http://www.cbioportal.org/) to analyse the alterations and correlations of ZBTB1 to explore its potential mechanism in tumour cells.^7^ ZBTB1 was altered in 3 samples of 1033 patients with pancreatic carcinoma (0.7%) (Figure 1K). Coexpression analysis of Segara's datasets showed that SLC4A7 expression was highly correlated with ZBTB1 in pancreatic carcinoma (Figure 1J). SLC4A7 has been shown to be expressed at significantly higher levels in human pancreatic ductal carcinoma, and silencing SLC4A7 in mice slows pancreatic tumour growth.^8^ Clearly, ZBTB1 did not appear to be prone to mutations in pancreatic carcinoma that resulted in effects on its neighbouring genes. Figure 1L shows 38 proteins with direct or indirect interactions with ZBTB1 (string‐DB database) (Figure 1L). ARMCX2 was also identified by Xu et al.^9^ to be a very important regulator in gastric cancer^9^; and BTBD1 localized in cytoplasmic bodies is increased in cancer cells.^10^ ZBTB1 may contribute to tumour cell proliferation and apoptosis by regulating these proteins.

The functions of ZBTB1 and the genes significantly associated with ZBTB1 were predicted by analysing gene ontology (GO) and Kyoto Encyclopedia of Genes and Genomes (KEGG) with the Database for Annotation, Visualization and Integrated Discovery (DAVID) (https://david.ncifcrf.gov/). Figure 1M shows that proteasomal protein catabolic processes, ubiquitin ligase complexes and ubiquitin‐like protein transferase activities in pancreatic cancer are significantly regulated by ZBTB1 and related genes (Figure 1M). It is well known that the ubiquitination proteasome pathway is inextricably linked to cancer initiation, progression and prognosis. Twenty‐five pathways related to ZBTB1 and its associated genes in pancreatic carcinoma were identified by KEGG analysis (Figure 1N). In these pathways, autophagy, ubiquitin‐mediated proteolysis and endocytosis are involved in the development and pathogenesis of pancreatic carcinoma.

In summary, this study is the first to explore the expression and prognostic value of ZBTB1 in pancreatic carcinoma tissue with tissue microarray (TMA) technology, immunohistochemistry (IHC), GEPIA, Oncomine, Kaplan‐Meier Plotter, cBioPortal, String‐DB, DAVID and other databases. Our results suggest that the enhanced expression of ZBTB1 contributes significantly to pancreatic carcinogenesis. ZBTB1 may be a potential new candidate biomarker to identify additional therapeutic targets in the battle against.
